# Adding-on nivolumab to chemotherapy-stabilized patients is associated with improved survival in advanced pancreatic ductal adenocarcinoma

**DOI:** 10.1007/s00262-024-03821-3

**Published:** 2024-09-09

**Authors:** Shih-Hung Yang, Sung-Hsin Kuo, Jen-Chieh Lee, Bang-Bin Chen, Yan-Shen Shan, Yu-Wen Tien, Sz-Chi Chiu, Ann-Lii Cheng, Kun-Huei Yeh

**Affiliations:** 1 Department of Oncology, National Taiwan University Hospital, No. 7, Chung-Shan South Road, Taipei, 10002 Taiwan; 2 Graduate Institute of Oncology, National Taiwan University College of Medicine, Taipei, Taiwan; 3 Department of Pathology, National Taiwan University Hospital, Taipei, Taiwan; 4 Department of Medical Imaging and Radiology, National Taiwan University Hospital, Taipei, Taiwan; 5 Division of General Surgery, Department of Surgery, College of Medicine, National Cheng Kung University Hospital, National Cheng Kung University, Tainan, Taiwan; 6 Department of Surgery, National Taiwan University Hospital, Taipei, Taiwan; 7 PharmaEngine, Inc., Taipei, Taiwan

**Keywords:** Pancreatic cancer, Chemotherapy, Disease control, Add-on nivolumab, Prognosis

## Abstract

**Background:**

Immune checkpoint inhibitors (ICIs) are rarely used to treat advanced pancreatic ductal adenocarcinoma (PDAC) due to marginal efficacy.

**Patients and methods:**

This study included 92 consecutive patients diagnosed with advanced or recurrent PDAC who received nivolumab-based treatment. Univariate and multivariate analyses were used to identify prognostic factors. A control group of 301 patients with PDAC who achieved disease control with palliative chemotherapy but without ICIs was selected for comparison using propensity score matching (PSM).

**Results:**

The median overall survival (OS) since nivolumab treatment was 15.8 (95% confidence interval [CI], 12.5–19.0), 2.4 (95% CI 1.2–3.6), and 1.1 (95% CI 1.0–1.2) months in patients who received add-on nivolumab after achieving disease control with chemotherapy, in those who received concomitant nivolumab and chemotherapy without prerequisite confirmation of disease control, and in those who received nivolumab without concomitant chemotherapy, respectively (*P* < 0.001). After PSM, the median overall survival (OS) since initiation of the concomitant chemotherapy that achieved disease control was significantly longer (*P* = 0.026) in patients who received add-on nivolumab (19.8 months; 95% CI 14.5–25.1) than in those who received chemotherapy alone (13.8 months; 95% CI 10.8–16.9). The immune profiling of the tumors in resected patients revealed higher scores of CD8^+^ T cells to Tregs in patients with add-on nivolumab comparing to those who received chemotherapy alone.

**Conclusion:**

Adding-on nivolumab was associated with improved OS in patients with advanced PDAC who achieved disease control following chemotherapy.

**Supplementary Information:**

The online version contains supplementary material available at 10.1007/s00262-024-03821-3.

## Introduction

Owing to its increasing incidence and mortality rate, pancreatic ductal adenocarcinoma (PDAC) is anticipated to remain a major global burden of disease in the upcoming decade [1]. According to cancer-related statistics in the GLOBOCAN 2020 report, > 450,000 new cases and approximately the same number of deaths are attributed to pancreatic cancer [2]. First-line treatment for metastatic PDAC including multiagent cytotoxic regimens, such as gemcitabine plus nab-paclitaxel (G/nab-P) and FOLFIRINOX (oxaliplatin, irinotecan, 5-fluorouracil [5-FU], and leucovorin), are effective in improving survival [3, 4]. In the NAPOLI-1 trial, the combination of nanoliposomal irinotecan (nal-IRI), 5-FU, and leucovorin (NaFL) was superior to 5-FU and leucovorin in terms of overall survival (OS) in patients with metastatic PDAC with disease progression after gemcitabine-based therapy [5]. However, the 5-year OS rate in patients with PDAC remains 10% [6].

Limited clinical success has been achieved with immune checkpoint inhibitors (ICIs) in the treatment of advanced PDAC. Addition of anti-programmed cell death protein 1 (anti-PD-1) agents, such as nivolumab or pembrolizumab, to first-line G/nab-P for PDAC did not demonstrate obvious improvement in the response rate (RR) and OS comparing to chemotherapy alone [3, 7, 8]. Nevertheless, the disease control rate (DCR) achieved with the combination of ICIs and first-line chemotherapy for PDAC was > 60%, which exceeded the DCR achieved with chemotherapy alone [3, 7, 8]. A higher RR and DCR was achieved in a phase 1b study with the addition of sotigalimab (CD40 agonist) to first-line nivolumab plus G/nab-P [9]. In the phase 2 PRINCE trial, although the promising RR and median OS of the same combination were not observed, the high DCR was maintained [10]. The addition of motixafortide (CXCR4 antagonist) to pembrolizumab plus NaFL for PDAC resulted in a high DCR but marginal improvement of median PFS and OS compared to the outcomes of the NAPOLI-1 trial [5, 11, 12].

Given these findings, we hypothesized that ICIs would be beneficial for patients who have already achieved disease control with chemotherapy. Therefore, this study aimed to explore strategies for the effective treatment of advanced PDAC using nivolumab and identify key prognostic factors for survival.

## Materials and methods

### Patient selection

This study was approved by the Research Ethics Committee of National Taiwan University Hospital (REC. No. 202206104RINC). We identified consecutive patients diagnosed with pancreatic malignancy between January 2016 and March 2022 in our hospital. Patients with PDAC who were treated with palliative systemic therapy were included. The exclusion criteria were as follows: patients with pathological subtypes other than adenocarcinoma, those who only received a clinical diagnosis but had no available cytopathological data, those who were previously enrolled in clinical trials of ICIs, those lacking complete medical records or tumor response data, and those treated with ICIs other than nivolumab. Eligible patients were grouped as follows: the chemotherapy control group (Group A) comprised patients who achieved disease control (complete response [CR], partial response [PR], or stable disease [SD]) with palliative chemotherapy, but without nivolumab, and the nivolumab group (Group B) comprised patients treated with nivolumab (with or without chemotherapy). Group B was further divided into three subgroups according to the nivolumab treatment strategy: Group B1 (add-on group) wherein nivolumab was added to any line of concomitant chemotherapy regimen after disease control had been documented with imaging studies, Group B2 (concurrent group) wherein nivolumab was initiated concurrently or beyond with any line of chemotherapy without prerequisite imaging-documented disease control of the concomitant chemotherapy, and Group B3 (no chemotherapy group) wherein nivolumab was used as monotherapy or in combination with targeted therapy).

### Imaging evaluation

Computed tomography or magnetic resonance imaging was usually performed for initial tumor staging and response evaluation every 3 months. The initial tumor stage was determined according to the American Joint Committee on Cancer staging system (eighth edition). Tumor response was evaluated according to the revised version of the Response Evaluation Criteria in Solid Tumors (version 1.1). Disease control for any chemotherapy was defined as at least one imaging-documented CR, PR, or SD observed using pre-chemotherapy imaging as the baseline. Imaging studies performed before the initiation of nivolumab treatment served as the baseline for evaluating the tumor response to nivolumab. The spleen volume was estimated using a previously described method [13]. Based on our institutional practice, patients who had received at least 3 to 6 months of (neoadjuvant) chemotherapy and the tumor had achieved stable disease or partial response in the imaging study with decreased levels of CA 19–9 wound receive surgical exploration.

Details of the NanoString® assay and immunohistochemistry (IHC) are provided in the Supplementary methods.

### Statistical analysis

The cutoff date for data collection was December 31, 2023. Survival data were analyzed using the Kaplan–Meier method and log-rank test. Between-variable differences in time to treatment failure (TTF) and OS were analyzed using Cox proportional-hazards regression. Between-group differences in clinical variables were analyzed using the Chi-squared test or Fisher’s exact test. Baseline characteristics (before nivolumab treatment) with borderline significance (*P* < 0.1) in the univariate analysis were included in the multivariate analysis. For patients receiving nivolumab, OS_nivo_ was calculated from the initiation of nivolumab treatment to the day of death or last follow-up, whereas TTF_nivo_ was calculated from the initiation of nivolumab treatment to the day of disease progression (confirmed through imaging studies), clinical progression, treatment intolerance, death, or last follow-up. For patients receiving chemotherapy with or without nivolumab, OS_chemo_ was calculated from the initiation of the corresponding chemotherapy regimen that achieved disease control to the day of death or last follow-up, whereas TTF_chemo_ was calculated from the initiation of the corresponding chemotherapy regimen that achieved disease control to the day of disease progression (confirmed through imaging studies), clinical progression, treatment intolerance, death, or last follow-up.

Propensity score matching (PSM) (1:1; nearest neighbor method) was performed according to the patients’ baseline characteristics before each line of chemotherapy (i.e., first, second, and subsequent chemotherapy) to compare disease prognosis between Groups A and B1. Details of the PSM and lymphocyte-neutrophil ratio (LNR) analyses are provided in the Supplementary methods.

Statistical analyses were performed using SPSS for Windows (version 20.0; IBM Corp., Armonk, NY, USA). *P* < 0.05 was considered statistically significant.

## Results

### Demographics of the nivolumab-treated patients

Of the 1,872 initially screened patients, 301 and 92 constituted the chemotherapy control group (Group A) and the nivolumab group (Group B), respectively. Group B was further subdivided into groups B1 (*n* = 43), B2 (*n* = 33), and B3 (*n* = 16) according to the nivolumab treatment strategy (Supplementary Fig. 1). Baseline characteristics of the patients in Group B are summarized in Table 1. The median interval from the initiation of first-line palliative chemotherapy to the initiation of nivolumab treatment was 6.2 (95% confidence interval [CI], 2.7–9.7), 10.8 (95% CI 7.3–14.2), and 4.2 (95% CI 1.4–7.0) months for groups B1, B2, and B3, respectively. The median interval from the initiation of concomitant chemotherapy to the addition of nivolumab treatment in Group B1 was 3.9 (95% CI, 3.1–4.7) months.

**Table 1:** Characteristics of patients before nivolumab treatment

Characteristics	Group B1		Group B2	Group B3	P
N	43	33	16		
Age (y/o)	median	65	62	62	0.783^†^
	range	37–78	46–81	52–73	
Sex	male	30	20	7	0.184
	female	13	13	9	
Stage at diagnosis	I	1	1	1	0.832
	II	5	7	2	
	III	8	7	2	
	IV	29	18	11	
ECOG PS at nivolumab start	0–1	38	11	1	< 0.001
	≥ 2	5	22	15	
Primary site in pancreas	head	26	17	6	0.183
	body	6	11	6	
	tail	11	5	4	
Curative surgery	Yes	7	11	3	0.195
	No	36	22	13	
Radiotherapy to primary site	Yes	3	3	1	0.919
	No	40	30	15	
Prior palliative chemotherapy regimens	0	0	1	0	0.010
	1	19	3	4	
	2	11	6	3	
	> 2	13	23	9	
Prior used chemotherapy agents	Gem	41	32	15	0.867
	F	35	31	14	0.273
	Pt	26	28	11	0.068
	Pac	25	26	9	0.123
	Iri	16	26	9	0.001
Prior regimens with disease control	0	0	13	12	< 0.001
	1	33	13	4	
	2	10	7	0	
Locoregional tumor^#^ at nivolumab start	Yes	36	27	16	0.198
	No	7	6	0	
Metastasis at nivolumab start	Yes	33	31	16	0.021
	No	10	2	0	
Metastatic organ at nivolumab start	Liver	21	26	10	0.029
	Peritoneum	13	19	8	0.049
	Lung	7	10	9	0.010
Spleen volume at nivolumab start (ml)^§^	median	206	313	241	0.021^†^
	range	119–695	149–724	107–789	
MSI	High	0	1	0	NA
	Stable	23	10	2	
	Not tested	20	22	14	

ECOG PS, Eastern cooperative oncology group performance status; F, 5-FU/5-FU analog; Gem, gemcitabine; Iri, (liposomal) irinotecan; MSI, microsatellite instability; NA, not analyzed; Pac, (nab)-paclitaxel; Pt, platinum (oxaliplatin or cisplatin)

†Analysis of variance

§Excluding patients who underwent splenectomy

#Either initially unresectable local tumor or local recurrence in patients receiving curative surgery

### Nivolumab-based regimens

The nivolumab-based regimens and their associated outcomes are summarized in Table 2. Nivolumab ≥ 2.5 mg/kg/dose was administered to 16 (37%), 14 (42%), and 6 (38%) patients in Groups B1, B2, and B3, respectively (*P* = 0.889) in a biweekly schedule. In Group B1, 13 patients received add-on cytokine-induced killer (CIK) cell therapy after a median of 7 (range, 0–14) doses of nivolumab. Notably, after achieving a partial response to the add-on nivolumab and chemotherapy, patients in Group B1 underwent resection of the primary tumor (*n* = 1), liver metastases (*n* = 1), or both (*n* = 1) with conversion surgery.

**Table 2:** Regimens used concomitantly with nivolumab (NIVO) and their outcomes

Group	N	Dose of NIVO^§^ (mg/kg)	Concomitant agents		Response				Resection after NIVO	OS after NIVO (month)	OS after first-line chemo (month)
			CIK (N)	Regimen for combination	N	CR/PR/SD	PD	NA			
B1	43	2.0 0.3–3.0	13	Nal/HDFL	8	1/3/28	6	5	3	15.8 (12.5–19.0)	23.0 (18.3–27.6)
				GN	7						
				SLOG	5						
				SLOG/RT	3						
				GN/RT, GNSL, GS, SOLAR, SOLAR/RT	2						
				CySL, FOLFIRINOX, GNCy/RT, GNS/RT, GOS, GOSCy, Nal/HDFL/RT, Nal/HDFL/Tra, NALIRIFOX, NI/HDFL	1						
B2	33	2.3 0.3–4.0	1	GN	4	1/0/0	18	14	0	2.4 (1.2–3.6)	15.8 (13.4–18.2)
				Nal/HDFL, SOLAR	3						
				GO, M/HDFL, NI/HDFL, SLCG	2						
				FOLFIRINOX, G, GOFL, HDFL, I/HDFL, ICap, IOS, Nal/HDFL/Bev, Nal/S, NIS, NMF, NMS, OCap, SLOG, SOLARCy	1						
B3	16	2.3 0.5–3.4	1	None	14	0/1/0	3	12	0	1.1 (1.0–1.2)	6.2 (0–16.2)
				Regorafenib	2						

Bev, bevacizumab; C, cisplatin; Cap, capecitabine; CIK, cytokine-induced killer (cell therapy); CR, complete response; Cy, cyclophosphamide; F, 5-FU; G, gemcitabine; HDFL, high-dose 5-FU/leucovorin; I, irinotecan; L, leucovorin; M, mitomycin C; N, nab-paclitaxel; NA, not analyzed; Nal, nanoliposomal irinotecan; O, oxaliplatin; OS, overall survival (median & 95% confidence interval); PD, progressive disease; PR, partial response; RT, radiotherapy to primary site; S, S-1; SD, stable disease; Tra, trametinib

FOLFIRINOX: irinotecan, oxaliplatin, 5-FU, and leucovorin; NALIRIFOX: nanoliposomal irinotecan, oxaliplatin, 5-FU, and leucovorin; SOLAR: nab-paclitaxel, oxaliplatin, S-1, and leucovorin

§Dose of NIVO (mg/kg): median and range

### Prognosis and outcomes of the nivolumab-treated patients

**Fig. 1:**
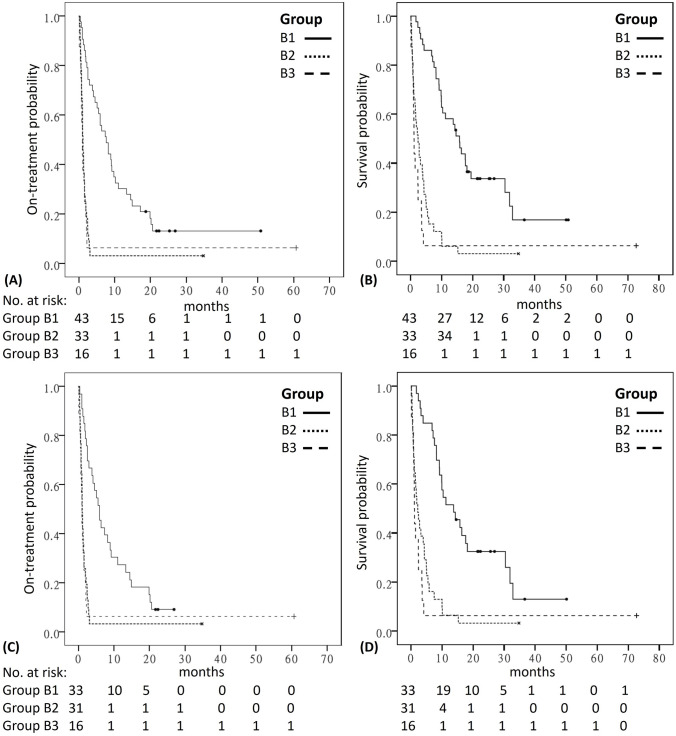
Kaplan–Meier plots and at-risk tables for the comparison of **A** TTF and **B** OS among all patients who received nivolumab (Group B) and for the comparison of **C** TTF and **D** OS among patients who had distant metastasis before receiving nivolumab. OS, overall survival; TTF, time to treatment failure. TTF and OS were calculated since the initiation of nivolumab treatment. (Group B1, add-on group; Group B2, concurrent group; Group B3, no chemotherapy Group)

Considering the imaging study before nivolumab treatment as the baseline to evaluate the response, the overall RR was 9% (4/43), 3% (1/33), and 6% (1/16) for groups B1, B2, and B3, respectively. The median TTF_nivo_ was 7.6 (95% CI 4.7–10.5), 1.1 (95% CI 0.9–1.4), and 0.9 (95% CI 0.7–1.1) months for groups B1, B2, and B3, respectively (*P* < 0.001; Fig. 1A). The median OS_nivo_ was 15.8 (95% CI 12.5–19.0), 2.4 (95% CI 1.2–3.6), and 1.1 (95% CI 1.0–1.2) months for groups B1, B2, and B3, respectively (*P* < 0.001; Fig. 1B). The median OS since the initiation of first-line palliative chemotherapy was 23.0 (95% CI 18.3–27.6), 15.8 (95% CI 13.4–18.2), and 6.2 (95% CI 0–16.2) months for groups B1, B2, and B3, respectively (*P* < 0.001).

Considering patients with distant metastasis at the initiation of nivolumab treatment (*n* = 80), the median TTF_nivo_ for Groups B1, B2, and B3 was 6.0 (95% CI 3.9–8.0), 1.0 (95% CI 0.9–1.2), and 0.9 (95% CI 0.7–1.1) months (*P* < 0.001; Fig. 1C), and the median OS_nivo_ was 13.7 (95% CI 7.3–20.2), 2.2 (95% CI 0.8–3.5), and 1.1 (95% CI 1.0–1.2) months (*P* < 0.001; Fig. 1D), respectively. The median OS since the initiation of first-line palliative chemotherapy was 20.9 (95% CI 14.3–27.6), 15.8 (95% CI 13.4–18.1), and 6.2 (95% CI 0–16.2) months for groups B1, B2, and B3, respectively (*P* = 0.001).

The median TTF and OS since the initiation of G/nab-P in Group B1 patients with initial stage IV disease receiving add-on nivolumab following disease control with first-line G/nab-P (*n* = 7) were 9.0 (95% CI 3.5–14.4) months and not reached (> 19.8 months), respectively; the prognosis since the initiation of the concomitant chemotherapy was even better in Group B1 patients receiving first-line triplet regimens (*n* = 5) with the median TTF of 25.5 (95% CI 3.8–47.2) months and the median OS of 42.6 (95% CI, 8.8–76.3) months. The median TTF and OS since the initiation of NaFL in Group B1 patients with distant metastasis who failed prior gemcitabine-based treatment and received add-on nivolumab after achieving disease control with NaFL (*n* = 10) were 10.6 (95% CI, 8.2–13.1) and 16.5 (95% CI, 10.7–22.4) months, respectively.

In Group B1, 13 (30%) patients were treated with concomitant CIK cell therapy. Among them, in patients with (*n* = 13; 10 of them with metastatic disease) and without (*n* = 30; 23 of them with metastatic disease) CIK cell therapy, the median TTF since the initiation of the concomitant chemotherapy achieving disease control was 22.9 (95% CI 7.1–38.7) months and 11.4 (95% CI 7.8–15.1) months, respectively (*P* = 0.135); the median TTF_nivo_ was 14.4 (95% CI 8.8–20.0) months and 6.0 (95% CI 2.4–9.5) months, respectively (*P* = 0.120).

In addition to nivolumab, responders in Group B1 received gemcitabine, oxaliplatin, S-1, leucovorin (*n* = 2), or G/nab-P (*n* = 2; 1 with concomitant radiotherapy). The responder in Group B2, who received gemcitabine, cisplatin, and S-1 with nivolumab, had microsatellite instability (MSI)-high tumors with a high tumor mutation burden (TMB) and germline *MSH6* mutation. In contrast, the responder in Group B3, who received nivolumab monotherapy after the failure of first-line S-1, harbored *PBRM1* and *POLE* mutations.

Prominent differences were noted in baseline characteristics between the B1 and B2 groups (Supplementary Table 1). Univariate and multivariate analyses, which included unbalanced baseline characteristics, revealed that the strategy of add-on nivolumab, absence of liver metastasis, and an ECOG performance status of 0–1 at the initiation of nivolumab treatment were independent and good prognostic factors for OS_nivo_ and TTF_nivo_ (Table 3).

**Table 3:** Prognosis analyses for nivolumab-chemotherapy combination therapy

Parameter value	Overall survival		Time to treatment failure	
	Univariate	Multivariate	Univariate	Multivariate
	OR (95% CI) P1^*^	OR (95% CI) P2^*^	OR (95% CI) P1^*^	OR (95% CI) P2^*^
ECOG PS at nivolumab start 0–1 vs ≥ 2	0.06 (0.03–0.12)	0.05 (0.02–0.12)	0.14 (0.08–0.25)	0.16 (0.08–0.32)
	< 0.001	< 0.001	< 0.001	< 0.001
Prior palliative regimens 0–2 versus ≥ 3 lines	0.32 (0.19–0.53)	0.76 (0.40–1.47)	0.42 (0.26–0.69)	0.69 (0.36–1.32)
	< 0.001	0.421	0.001	0.263
Prior platinum no versus yes	0.38 (0.21–0.70)	0.50 (0.25–1.00)	0.63 (0.37–1.07)	0.85 (0.47–1.54)
	0.002	0.051	0.087	0.582
Prior (liposomal) irinotecan no versus yes	0.30 (0.17–0.50)	0.47 (0.23–0.95)	0.41 (0.25–0.67)	0.89 (0.45–1.78)
	< 0.001	0.034	< 0.001	0.741
Liver mets at nivolumab start no versus yes	0.52 (0.31–0.89)	0.41 (0.22–0.77)	0.50 (0.30–0.82)	0.53 (0.30–0.93)
	0.016	0.006	0.007	0.026
Peritoneal mets at nivolumab start (no versus yes)	0.73 (0.44–1.21)		0.91 (0.56–1.47)	
	0.226		0.696	
Spleen volume^§^ < 200 ml versus ≥ 200 ml	0.57 (0.34–0.96)	0.69 (0.40–1.20)	0.89 (0.55–1.44)	
	0.034	0.191	0.639	
Nivolumab strategy add-on vs concurrent	0.21 (0.12–0.36)	0.51 (0.26–0.98)	0.22 (0.13–0.37)	0.42 (0.22–0.83)
	< 0.001	0.044	< 0.001	0.012

CI, confidence interval; ECOG PS, eastern cooperative oncology group performance status; OR, odds ratio

*P1 and P2: between-variable differences (Cox regression)

§Patients who underwent splenectomy were included in the subgroup with a spleen volume of < 200 mL

Among the 79 patients who had treatment failure of the nivolumab-based treatment at the end of follow-up, 40 patients received subsequent therapy, including (liposomal) irinotecan, nab-paclitaxel, or platinum-based regimens in 32 patients, gemcitabine-based ones in six patients, and others in two patients, respectively.

### PSM—chemotherapy with/without nivolumab

Baseline characteristics before the first palliative chemotherapy regimen that achieved disease control in Group A are summarized in Supplementary Table 2. Details of the chemotherapy regimens used in Group A are summarized in Supplementary Table 3. The median OS since the initiation of first-line palliative chemotherapy was 15.9 months (95% CI 14.5–17.3) for all patients in Group A and 14.2 months (95% CI 12.7–15.6) for those with the initially stage IV disease (*n* = 143). Baseline characteristics of Groups B1 and A stratified by the line of chemotherapy were well balanced after PSM (Supplementary Table 4). The RR of chemotherapy achieving disease control without add-on nivolumab when comparing groups B1 and A (B1:20%; A: 15%; *P* = 0.357). The median interval from the first-line therapy to the initiation of the chemotherapy that had achieved disease control was 1.9 (range, 0–6.5) months and 2.2 months (range, 0.5–17.8) months in the second-line matching and 5.7 (range, 2.3–15.6) months and 8.8 (range, 2.7–35.6) months in the subsequent-line matching for Group B1 and Group A, respectively.

**Fig. 2:**
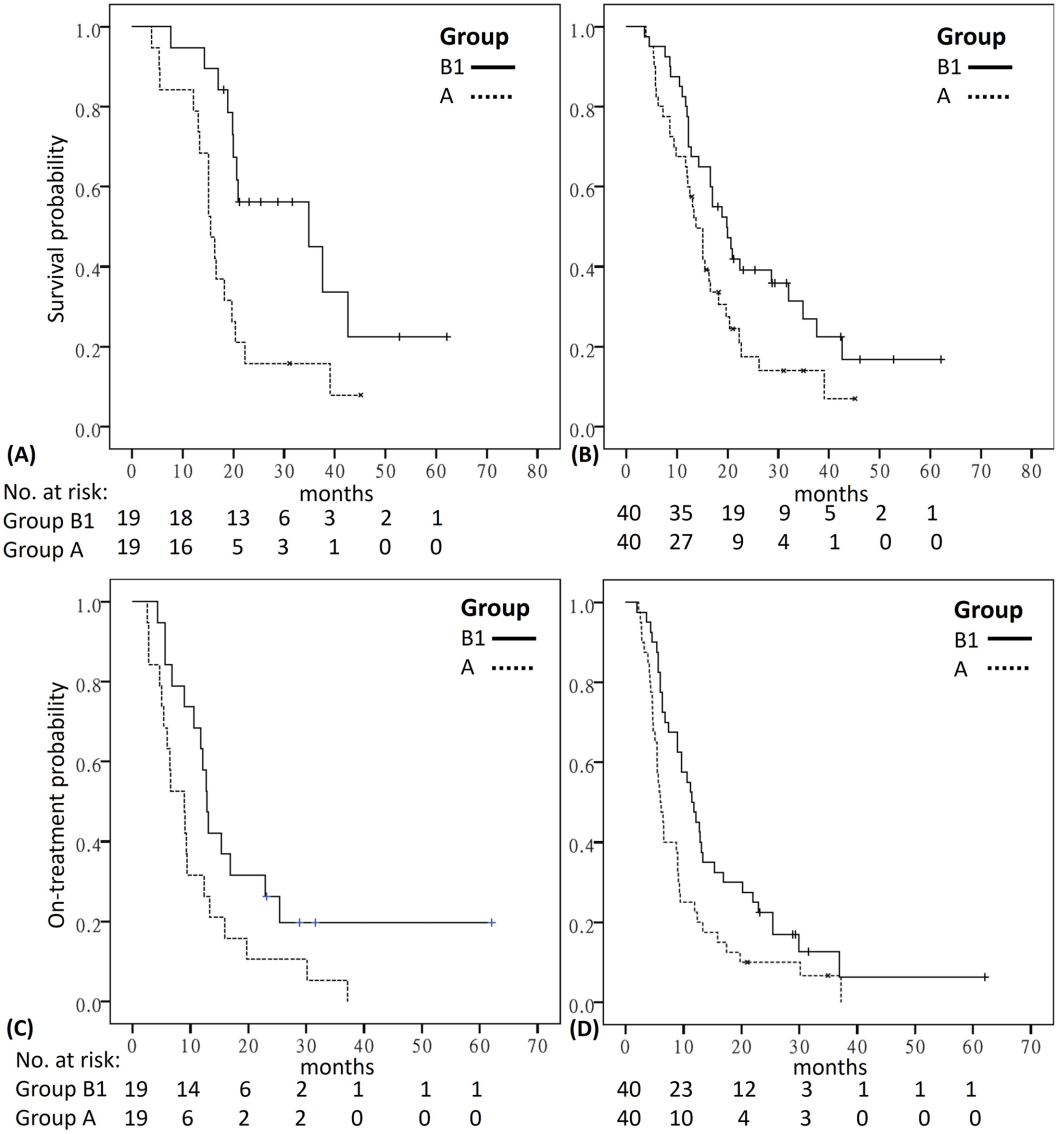
Kaplan–Meier plots and at-risk tables comparing OS between matching Group B1 and Group A in **A** patients of the first-line treatment and in **B** all patients and comparing TTF between matching Group B1 and Group A in **C** patients of the first-line treatment and in **D** all patients. OS, overall survival; TTF, time to treatment failure. (Group A, chemotherapy control group; Group B1, add-on group)

In the first-line matching (*n* = 38), the median OS_chemo_ was significantly better in Group B1 (34.9 months; 95% CI 1.5–68.2) compared to Group A (15.4 months; 95% CI 13.7–17.2) (*P* = 0.006, Fig. 2A). Overall (*n* = 80), the median OS_chemo_ was significantly longer for Group B1 than for propensity score-matched Group A (19.8 [95% CI 14.5–25.1] and 13.8 [95% CI 10.8–16.9] months, respectively; *P* = 0.026; Fig. 2B). The timing of adding-on nivolumab and changes of regimens in each case in first-line, second-line, and subsequent-line matching in Group B1 and Group A are demonstrated in Supplementary Fig. 2. Regarding the median TTF_chemo_, significant difference was noted in the first-line (B1 vs. A: 12.9 months vs. 9.0 months, *P* = 0.044; Fig. 2C) and subsequent-line (B1 vs. A: 8.9 months vs. 5.5 months, *P* = 0.015) but not second-line (B1 vs. A: 9.6 months vs. 5.4 months, *P* = 0.587) matching. Overall (*n* = 80), the median TTF_chemo_ was significantly longer in Group B1 than in propensity score-matched Group A (11.4 [95% CI 9.1–13.8] and 6.0 [95% CI 4.7–7.3] months, respectively; *P* = 0.013; Fig. 2D). The estimated E-value of add-on nivolumab was 2.98 and 2.92 for TTF_chemo_ and OS_chemo_, respectively. The favorable trend of survival in Group B1 over Group A was maintained in patients without CIK therapy after PSM and was similar in the data irrespective of CIK therapy (Supplementary Table 5 & 6).

### Profiling of immune cells and pathways in tumor tissues

**Fig. 3:**
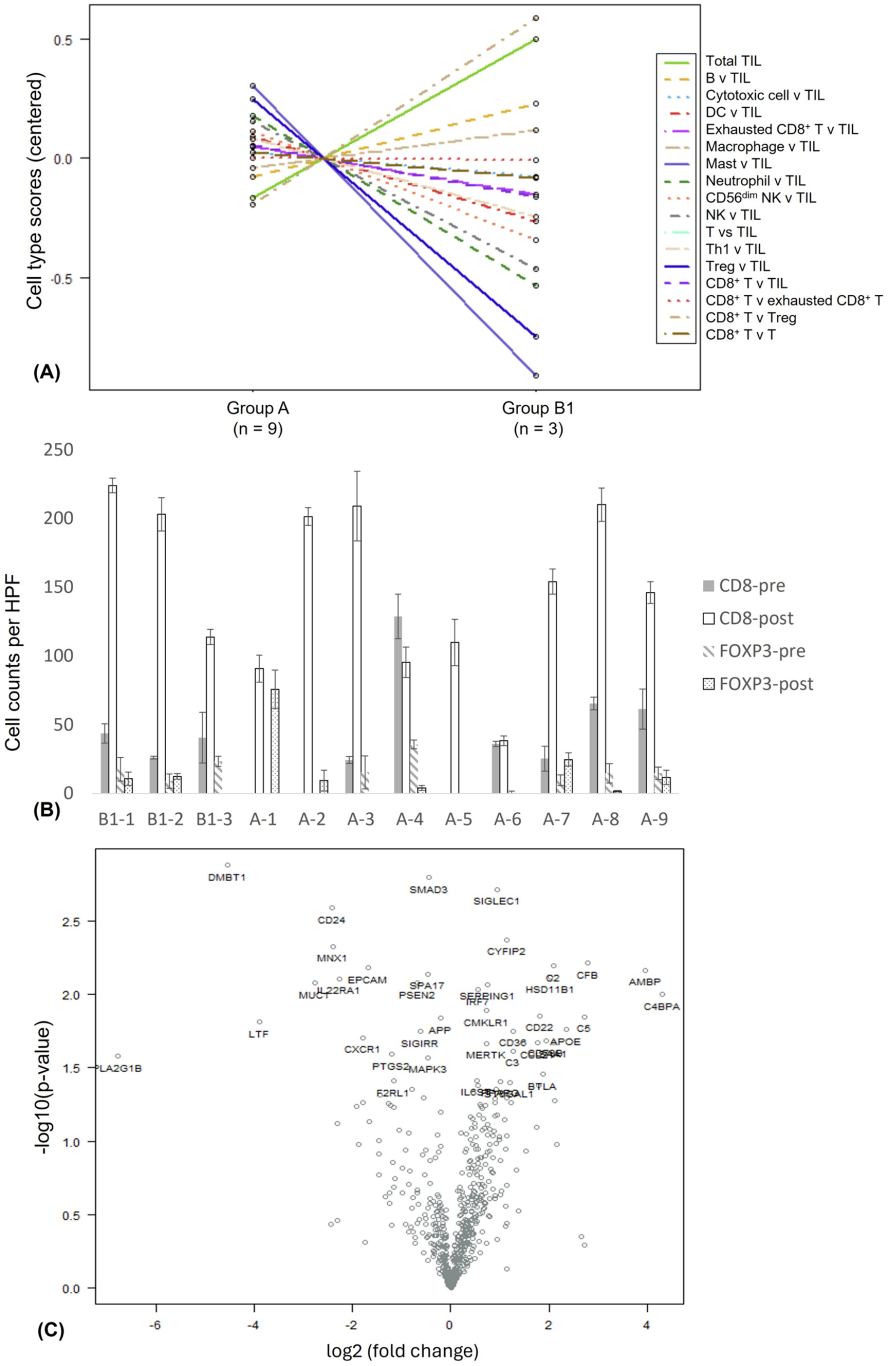
The resected tumors from patients receiving conversion surgery after chemotherapy ± nivolumab showing **A** the relative ratio of immune cells in the tumor microenvironment (TME) analyzed by the NanoString® assay comparing Group B1 (*n* = 3) to Group A (*n* = 9); **B** the counts of immunohistochemical staining CD8^+^ cells and FOXP3^+^ cells in the TME of Group B1 (*n* = 3) and Group A (*n* = 9) before (pre-)^*^ and after (post-) chemotherapy ± nivolumab; **C** the relative RNA expression of the TME analyzed by the NanoString® assay comparing Group B1 (*n* = 3) to Group A (*n* = 9). (Group A, chemotherapy control group; Group B1, add-on group); ^*^The pre-chemotherapy biopsy samples from patient A1, A2, and A5 were not available due to scarce archival tissues

Nearly half of the patients underwent genetic testing for tumor tissues (Supplementary Table 7). Among the 12 patients who underwent resection of the primary tumor and/or liver metastasis (Supplementary Table 8), nine were from Group A and the other three were from Group B1. The post-chemotherapy surgical specimens, but not the pre-chemotherapy biopsied specimens, were adequate for mRNA-based profiling of immune cells and molecules. Regarding the analysis of cell type measurement in the post-chemotherapy tumor bed, the most obvious relative difference between the two groups was the high ratio of CD8^+^ T cells to Tregs in Group B1, in contrast, the ratio of neutrophils to TILs was low in Group B1 (Fig. 3A). IHC staining for CD8 and FOXP3 in the same post-chemotherapy tumor bed and pre-chemotherapy biopsied samples was performed for nine patients (three from Group B1 and six from Group A) with adequate archival tissues. The IHC staining data of the post-chemotherapy samples were compatible with the RNA-based profiling of immune cells. All three patients from Group B1 had a much higher number of CD8^+^ T cells after chemotherapy plus nivolumab, compared with four of the six patients in Group A after chemotherapy alone (Fig. 3B). C4BPA and PLA2G1B showed the most positive (19.8) and negative (0.009) fold changes in gene expression, respectively, when comparing Group B1 differentially expressed mRNA in the post-chemotherapy tumor bed to Group A (Fig. 3C, Supplementary Table 9).

## Discussion

In the present study, we performed retrospective analyses for the outcomes of patients with advanced PDAC treated with nivolumab-based therapy. In contrast to previous clinical trials utilizing ICIs, we first demonstrated clinical benefits in terms of OS and TTF with add-on nivolumab in patients achieving imaging-documented disease control under preceding chemotherapy. To further confirm the survival benefit of the add-on nivolumab strategy, a control group of patients achieving disease control through palliative chemotherapy without ICIs were selected for comparison. The patients treated with the add-on nivolumab strategy and chemotherapy still had significantly better OS and TTF comparing to those with chemotherapy alone. Notably, we also evaluated the tumor tissues from 12 patients who underwent conversion surgery following responding to chemotherapy with or without nivolumab. The tumor microenvironment (TME) revealed dense lymphocyte infiltrates. In addition, the scores of CD8^+^ T cells to Tregs were higher comparing patients with add-on nivolumab to those without.

ICIs that block PD-1, PD ligand 1 (PD-L1), and cytotoxic T-lymphocyte-associated protein 4 (CTLA-4) exhibit limited efficacy against PDAC. In the KEYNOTE-028 study, no patient with PDAC responded to pembrolizumab [14]. Similarly, in another study, one patient exhibited a delayed response to ipilimumab [15]. Moreover, the combination of durvalumab and tremelimumab resulted in an RR of 3.1% with the median PFS of 1.5 months in patients with one first-line gemcitabine or 5-FU-treated advanced PDAC [16]. These findings are consistent with those of our study: the median TTF_nivo_ and OS_nivo_ for Group B3 were 0.9 and 1.1 months, respectively.

Only a few patients with PDAC exhibit ICI-responsive genetic alterations [17]. Therefore, the combination of nivolumab with GVAX (allogeneic pancreatic tumor cells secreting granulocyte–macrophage colony-stimulating factor) and CRS-207 (attenuated *Listeria monocytogenes* expressing mesothelin) was used to enhance the antitumor immune response to PDAC; however, the RR was only 2% [18]. In a previous study, patients with advanced PDAC who achieved disease control with front-line FOLFIRINOX were randomized to continued chemotherapy or GVAX with ipilimumab for maintenance therapy; the OS and PFS were significantly worse in the immunotherapy arm with an RR of 2.9% [21]. This highlighted the importance of chemotherapy for controlling systemic tumor growth in PDAC and the weak antitumor activity of ICIs plus vaccine against PDAC [19].

Neoantigen-reactive CD4^+^ or CD8^+^ tumor infiltrating T lymphocytes (TILs) have rarely been detected in PDAC [20]. Theoretically, chemotherapy may reshape antitumor immunity. The use of the G/nab-P regimen resulted in considerable depletion of the desmoplastic stroma and increased concentration of gemcitabine within the tumor [21]. The reduced stromal activation was also associated with increased dendritic cells, proportion of CD8^+^ TILs but reduced myeloid-derived suppressor cells and the proportion of CD4^+^ FOXP3^+^ regulatory T cells (Tregs) in TME [22]. Gemcitabine or paclitaxel upregulated the expression of major histocompatibility complex class I, PD-L1, and PD-L2 in PANC-1 cells [23]. In most patients who underwent tumor resection in our study, the number of CD8^+^ T cells in the TME increased after chemotherapy, irrespective of nivolumab use. The trend of increased LNR in the peripheral blood (Supplementary Fig. 3) was similar between groups B1 and A; however, the total TILs and the ratio of CD8^+^ T cells to FOXP3^+^ Tregs in the TME were higher in patients receiving add-on nivolumab (Group B1) than in those receiving chemotherapy (Group A). The TME that expressed high C4BPA but low PLA2G1B in Group B1 compared to Group A may partially explain the enhanced infiltration of TILs [24, 25].

Chemotherapy is the best approach for rapidly reducing the systemic tumor burden; individuals with rapid disease progression may obtain limited timely benefits from ICIs. For example, at least 20% of patients may exhibit cancer progression within 3 months of the initiation of first-line G/nab-P or FOLFIRINOX treatment; this proportion is approximately > 40% in patients receiving NaFL [3–5]. In our study (Supplementary Fig. 1), the number of patients (*n* = 340) who never achieved disease control with chemotherapy was higher than that of those who did (*n* = 301), whereas patients, who were not subjected to imaging evaluation (*n* = 156), might have experienced rapid clinical deterioration.

In the CCTG PA.7 phase II trial, wherein G/nab-P with durvalumab and tremelimumab was compared with G/nab-P alone in terms of efficacy against metastatic PDAC, the median OS, RR, and DCR were modestly, but not significantly, better in the G/nab-P/ICI arm than in the G/nab-P arm [26]. In contrast, in a phase I/II trial in patients with locally advanced or metastatic PDAC who had achieved disease control with at least 16 weeks of platinum-based chemotherapy, the RR was low for niraparib plus nivolumab or ipilimumab. However, the median OS of both arms was quite long compared to that of FOLFIRINOX [4, 27]. In our study, the favorable outcomes of add-on nivolumab to chemotherapy highlighted the impact of timing, disease status, and chemotherapy on achieving meaningful clinical benefits of ICI. The RR of add-on nivolumab was anticipated to be low (9%), but still higher than that of ICIs alone [16]. Nevertheless, the median TTF_chemo_ and OS_chemo_ were significantly longer in patients receiving add-on nivolumab after achieving disease control with chemotherapy than in those receiving nivolumab without prerequisite confirmation of disease control or those receiving chemotherapy alone for disease control. Moreover, the absolute improvement in TTF_chemo_ was approximately 4 months, regardless of the timing of add-on nivolumab.

We propose several reasons for the significant improvements in disease progression and survival after add-on nivolumab therapy. First, patients with rapid disease progression and aggressive tumor biology were excluded after chemotherapy preceding add-on nivolumab treatment because their condition may worsen earlier or progress more rapidly with initial concomitant use of ICIs. In the CCTG PA.7 phase II trial, the deterioration of physical function and global health status in weeks 8 and 16 was numerically, but not significantly, higher in the G/nab-P/ICI arm than in the G/nab-P arm [26]. Second, effective preceding chemotherapy may elicit durable antitumor responses in the TME, thereby facilitating the effects of subsequent ICI treatment [22, 23]. In patients undergoing tumor resection, CD4^+^ and CD8^+^ TILs increased in patients with neoadjuvant FOLFIRINOX compared to those with upfront surgery [28]. Similarly, a recent study evaluating TME after neoadjuvant therapy demonstrated that TILs increased in patients with CR, PR, or SD [31]. Third, chemotherapy reduced or stabilized the tumor load. A small tumor burden has been demonstrated to predict the efficacy of ICIs for non-small-cell lung cancer [30]. Finally, in the absence of effective preceding chemotherapy, the detrimental effects of the specialized TME in PDAC may offset the benefit of the initial combination of ICI and chemotherapy [31, 32].

Nonetheless, our study had some limitations. The dose and schedule of the chemotherapy regimens were not consistent among patients treated with or without nivolumab. Moreover, the dose and timing of the nivolumab treatment were inconsistent, reflecting a real-world scenario. Information on the immune phenotypes of peripheral blood mononuclear cells was unavailable. Thus, the most effective regimen and optimal timing for add-on nivolumab treatment remain unknown. Patients, who received ICIs other than nivolumab, were excluded from this study. The fact that adding-on nivolumab was associated with prolonged survival may be partially explained by the immortal-time bias in this retrospective study. However, the difference of median OS_chemo_ between Group B1 and Group A was 19.5 and 6.0 months in the first-line and overall comparisons, respectively. The median interval from the initiation of first-line chemotherapy to adding-on nivolumab was 4.0 months in the first-line matching of Group B1. Although the immortal-time bias indeed existed, the large differences of median OS_chemo_ between Group B1 and Group A after matching, especially for the first-line matching, may not be explained by the immortal-times bias only.

In summary, our study provides evidence for the favorable prognostic implications and therapeutic efficacy of nivolumab in advanced PDAC that achieved disease control with preceding chemotherapy. Further exploration using nivolumab, such as the exploration of the adding-on strategy in patients achieving response or control of CA 19–9 after preceding chemotherapy (ClinicalTrials.gov identifier: NCT04377048), or other immune checkpoint modulators in combination with an adequate chemotherapy backbone through clinical trials and basic studies is required to confirm the results of this retrospective hypothesis-generating study.

## Supplementary Information

Below is the link to the electronic supplementary material.

Supplementary file1 (JPG 639 KB)

Supplementary file2 (JPG 810 KB)

Supplementary file3 (JPG 274 KB)

Supplementary file4 (DOCX 21 KB)

Supplementary file5 (DOCX 23 KB)

Supplementary file6 (DOCX 23 KB)

Supplementary file7 (DOCX 20 KB)

Supplementary file8 (DOCX 30 KB)

Supplementary file9 (DOCX 28 KB)

Supplementary file10 (DOCX 18 KB)

Supplementary file11 (DOCX 18 KB)

Supplementary file12 (DOCX 19 KB)

Supplementary file13 (CSV 5 KB)

## Data Availability

The data that supported this article will be made available after the approval of REC and corresponding author under reasonable request.
