# Supplementary figures and images for: Adding-on nivolumab to chemotherapy-stabilized patients is associated with improved survival in advanced pancreatic ductal adenocarcinoma
Source: Cancer Immunol Immunother. 2024 Sep 9;73(11):227. doi: 10.1007/s00262-024-03821-3 (PMC11383886; doi:10.1007/s00262-024-03821-3)

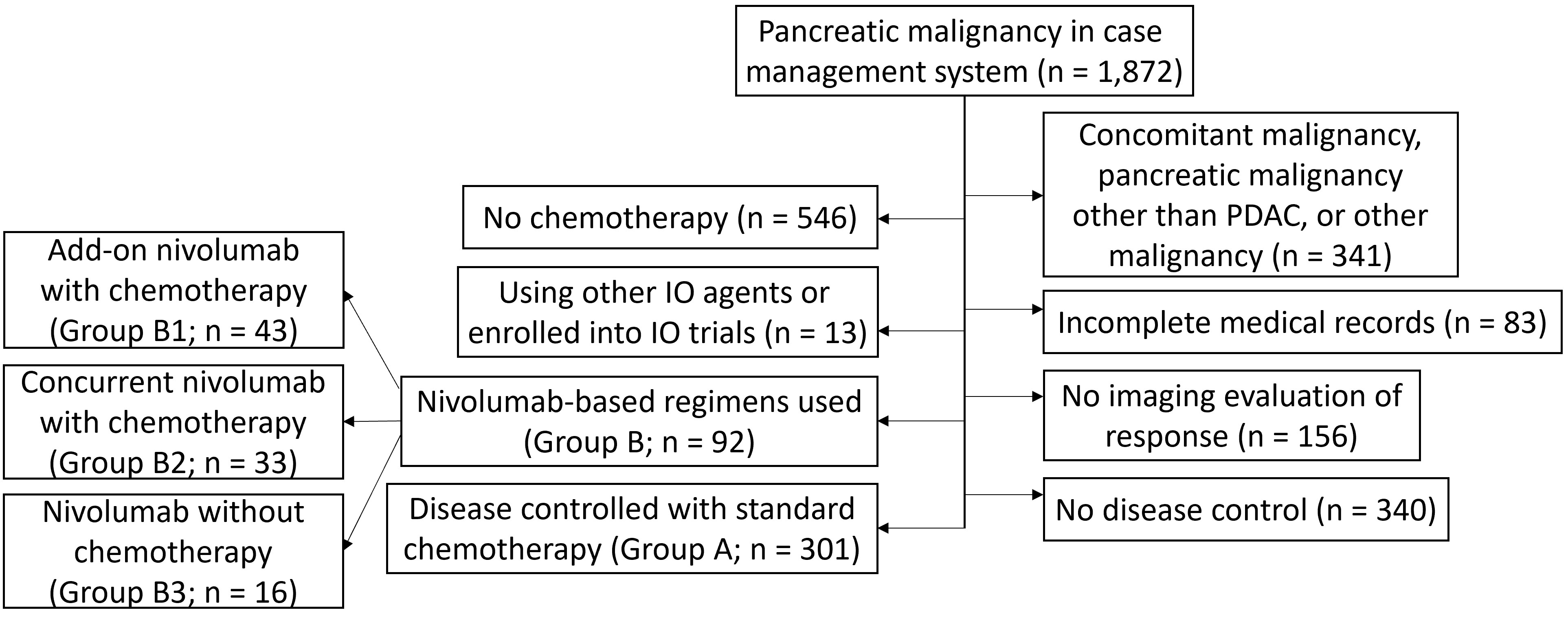

Supplement: Supplementary file 1 — Supplementary file1 (JPG 639 KB) [file 262_2024_3821_MOESM1_ESM.jpg]

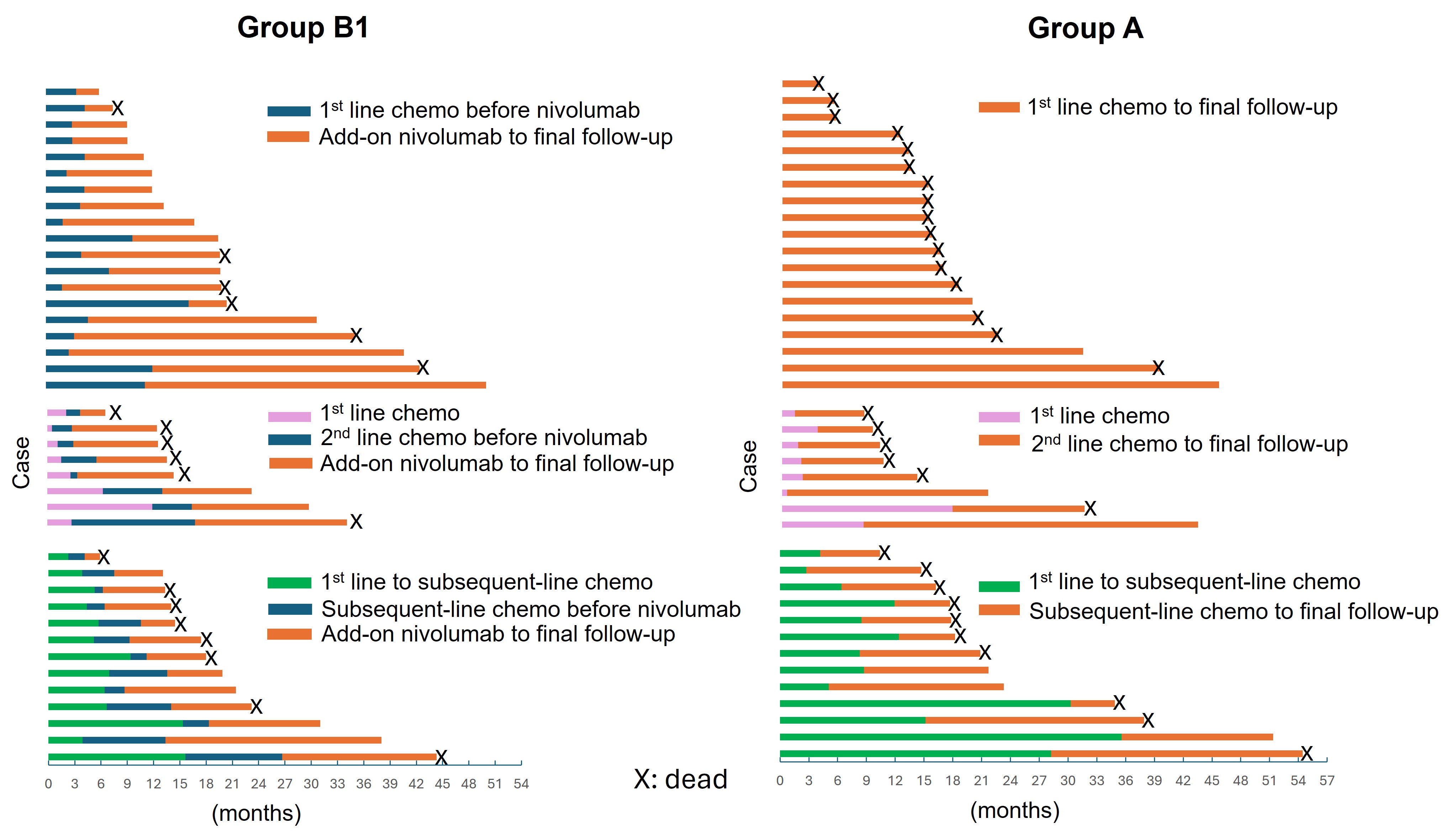

Supplement: Supplementary file 2 — Supplementary file2 (JPG 810 KB) [file 262_2024_3821_MOESM2_ESM.jpg]

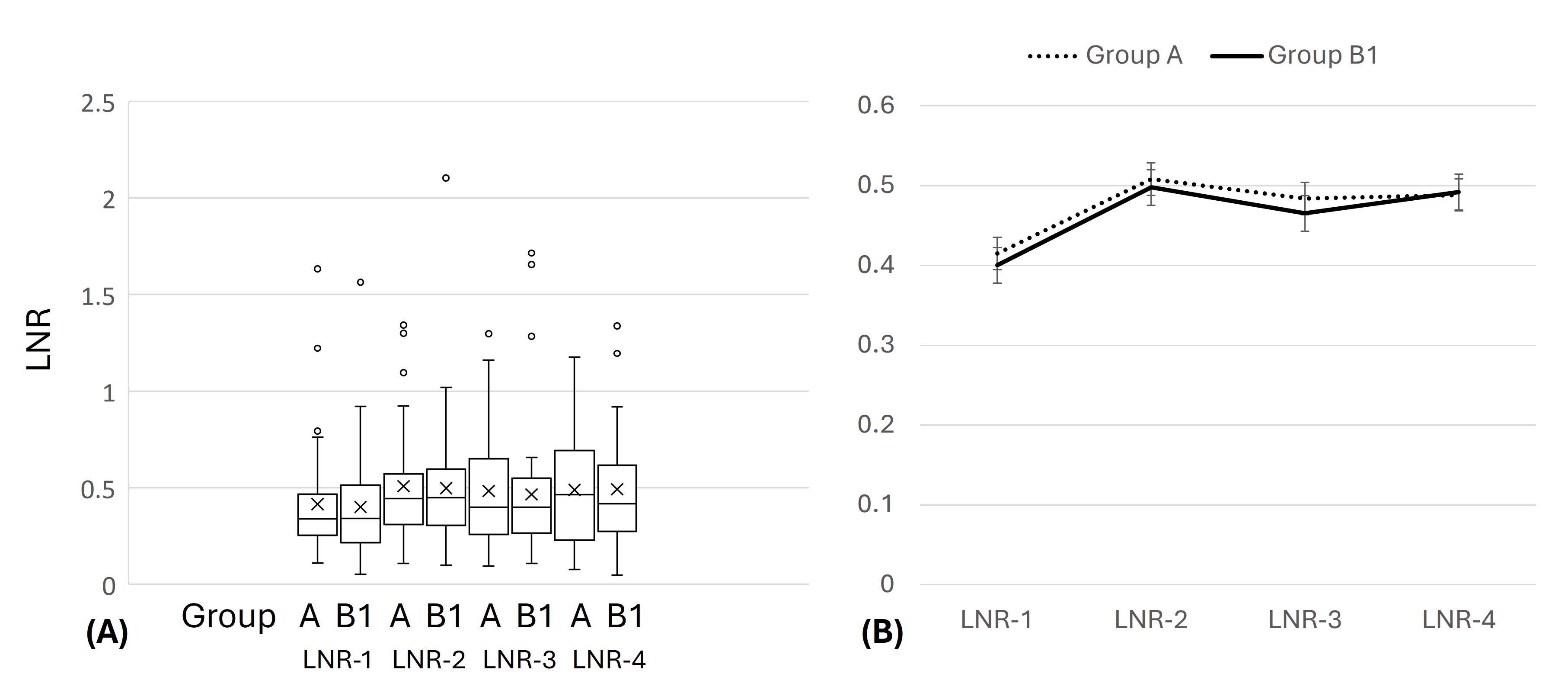

Supplement: Supplementary file 3 — Supplementary file3 (JPG 274 KB) [file 262_2024_3821_MOESM3_ESM.jpg]
