# Supplementary material for: Adding-on nivolumab to chemotherapy-stabilized patients is associated with improved survival in advanced pancreatic ductal adenocarcinoma
Source: Cancer Immunol Immunother. 2024 Sep 9;73(11):227. doi: 10.1007/s00262-024-03821-3 (PMC11383886; doi:10.1007/s00262-024-03821-3)
Supplement: Supplementary file 4 — Supplementary file4 (DOCX 21 KB) [file 262_2024_3821_MOESM4_ESM.docx]

**SUPPLEMENTARY METHODS**

**NanoString^®^ assay**

A commercial multiplexed gene expression panel, the NanoString nCounter PanCancer Immune Profiling Panel (NanoString Technologies, Seattle, WA, USA), was applied. Information of the gene list can be requested from the official website: at <https://nanostring.com/products/ncounter-assays-panels/oncology/pancancer-immune-profiling/>. In brief, we selected the region containing the highest densities of neoplastic ducts and infiltrated cancer cells according to the evaluation of the hematoxylin and eosin slides. Then, macrodissection was performed in 10–15 unstained slides of 10 μm-thickness, formalin-fixed, paraffin-embedded (FFPE) specimens from the post-chemotherapy surgical specimens. The quality of extracted RNA was well controlled with ≥20% of RNA fragments greater than 300 nucleotides (DV300). The samples were conducted on a Nanostring nCounter FLEX system (NanoString Technologies), and the raw data were analyzed with the nSolver 4.0 analysis software (NanoString Technologies).

**Immunohistochemistry**

Immunohistochemical (IHC) staining to FFPE tumor tissue sections (4-μm thick) were performed after deparaffinized by the EZ prep (Ventana Medical System, Inc., Tucson, AZ, USA). The anti-CD8 (clone SP57) primary antibody (#790-4460, Ventana, Roche) was used with the OptiView DAB Detection Kit (Ventana Medical System, Inc., Tucson, AZ, USA). The anti-FOXP3 primary antibody (#BSB 6761, Bio SB) was applied following the manufacturer's instructions (1:100). Stained tissue sections were evaluated by a pathologist, who was blinded to the patients' clinical data, and then reviewed by an oncologist. Three tissue areas were selected from the same region as the macrodissection for NanoString^®^ analysis with the highest density for CD8^+^ or FOXP3^+^ tumor infiltrating lymphocytes. CD8^+^ or FOXP3^+^ cell counts per high-power field (400x) were manually counted in pre-chemotherapy biopsied and post-chemotherapy surgical specimens.

**Propensity score matching (PSM)**

Regarding the first-line matching, patients in Group B1 who received add-on nivolumab after achieving disease control from their first-line chemotherapy were selected as the indicator cases. Then, patients who had achieved disease control from their first-line chemotherapy were selected from Group A according to the propensity score of the indicator cases of Group B1. With the same method in the second-line and subsequent-line matching, patients in Group A who had been unselected in the preceding steps were used for matching in the following steps.

A logistic regression model that included baseline characteristics as covariates was constructed to calculate propensity scores. The acceptable score difference in each matched pair was ≤0.3 (1 standard error of Group B1). For the comparison of Group A and B1, OS_chemo_ was calculated from the initiation of chemotherapy for achieving disease control to the day of death or last follow-up; TTF_chemo_ was calculated from the initiation of chemotherapy for achieving disease control to the day of disease progression (confirmed through imaging studies), clinical progression, treatment intolerance, death, or last follow-up. E-values were estimated to examine the impact of unmeasured confounders on the outcomes of patients [1]. The hazard ratio of add-on nivolumab for TTF_chemo_ and OS_chemo_ in the Cox proportional-hazards regression model comparing between matched Group B1 and A were used for estimation of risk ratio and calculation of E-values [1].

Factors for the first-line matching included age, sex, stage at initial diagnosis, primary site in pancreas, ECOG PS, presence of local regional tumor, metastasis, metastatic organ (liver, peritoneum, lung), curative surgery, radiotherapy to primary site, prior used chemotherapy agents, and first-line regimen. Factors for the second-line matching included baseline factors in the previous step and certain factors changed before second-line therapy, such as ECOG PS before second-line therapy, presence of local regional disease, metastasis, metastatic organ (liver, peritoneum, lung), radiotherapy to primary site, prior used chemotherapy agents, first-line regimen, and second-line regimen. The same rules were applied for selecting factors for subsequent-line matching.

As for matching for patients without CIK cell therapy, the criteria for first-line matching were the same as previously described. The criteria for second-line matching also followed the previous ones except for first-line regimen and second-line regimen. No matching was performed for the subsequent lines due to small number of patients left.

**Lymphocyte-neutrophil ratio (LNR) analysis**

The LNR of peripheral blood represented the percentage of lymphocyte divided by the percentage of neutrophil. The LNR in Group B1 was evaluated before the regimen achieving disease control (LNR-1), before starting nivolumab (LNR-2), after two doses of nivolumab (LNR-3), and after four doses of nivolumab (LNR-4). For comparison, the respective time points [i.e., before the regimen achieving disease control (LNR-1), imaging-confirmed disease control (LNR-2), 6 and 10 weeks after imaging-confirmed disease control (LNR-3 and LNR-4)] in matched subjects of Group A were also arbitrarily selected.

**Reference**

1. VanderWeele TJ, Ding P (2017) Sensitivity Analysis in Observational Research: Introducing the E-Value. Ann Intern Med 167:268-274
