# Supplementary material for: Adding-on nivolumab to chemotherapy-stabilized patients is associated with improved survival in advanced pancreatic ductal adenocarcinoma
Source: Cancer Immunol Immunother. 2024 Sep 9;73(11):227. doi: 10.1007/s00262-024-03821-3 (PMC11383886; doi:10.1007/s00262-024-03821-3)
Supplement: Supplementary file 5 — Supplementary file5 (DOCX 23 KB) [file 262_2024_3821_MOESM5_ESM.docx]

**Supplementary Table 1 Characteristics of patients before nivolumab treatment**

| **Characteristics** | | **Group B1** | **Group B2** | **P** |
| --- | --- | --- | --- | --- |
| N | | 43 | 33 |  |
| Age (y/o) | median  range | 65  37-78 | 62  46-81 | 0.503^†^ |
| Sex | male  female | 30  13 | 20  13 | 0.404 |
| Stage at diagnosis | I  II  III  IV | 1  5  8  29 | 1  7  7  18 | 0.640 |
| ECOG PS at nivolumab start | 0-1  ≥2 | 38  5 | 11  22 | <0.001 |
| Primary site in pancreas | head  body  tail | 26  6  11 | 17  11  5 | 0.113 |
| Curative surgery | Yes  No | 7  36 | 11  22 | 0.083 |
| Radiotherapy to primary site | Yes  No | 3  40 | 3  30 | 1.000 |
| Prior palliative chemotherapy regimens | 0  1  2  >2 | 0  19  11  13 | 1  3  6  23 | 0.001 |
| Prior used chemotherapy agents | Gem  F  Pt  Pac  Iri | 41  35  26  25  16 | 32  31  28  26  26 | 1.000  0.172  0.020  0.058  <0.001 |
| Prior regimens with disease control | 0  1  2 | 0  33  10 | 13  13  7 | <0.001 |
| Locoregional tumor at nivolumab start | Yes  No | 36  7 | 27  6 | 0.827 |
| Metastasis at nivolumab start | Yes  No | 33  10 | 31  2 | 0.058 |
| Metastatic organ at nivolumab start | Liver  Peritoneum  Lung | 21  13  7 | 26  19  10 | 0.008  0.017  0.146 |
| Spleen volume^§^ | <200 ml  ≥200 ml | 14  23 | 3  23 | 0.024 |
| Splenectomy | Yes  No | 6  37 | 7  26 | 0.405 |
| MSI | High  Stable  Not tested | 0  23  20 | 1  10  22 | NA |

^†^t test.

^§^Excluding patients who underwent splenectomy.

ECOG PS, Eastern Cooperative Oncology Group performance status; F, 5-FU/5-FU analog; Gem, gemcitabine; Iri, (liposomal) irinotecan; MSI, microsatellite instability; NA, not analyzed; Pac, (nab)-paclitaxel; Pt, platinum (oxaliplatin or cisplatin)
