# Supplementary material for: Adding-on nivolumab to chemotherapy-stabilized patients is associated with improved survival in advanced pancreatic ductal adenocarcinoma
Source: Cancer Immunol Immunother. 2024 Sep 9;73(11):227. doi: 10.1007/s00262-024-03821-3 (PMC11383886; doi:10.1007/s00262-024-03821-3)
Supplement: Supplementary file 6 — Supplementary file6 (DOCX 23 KB) [file 262_2024_3821_MOESM6_ESM.docx]

**Supplementary Table 2 Baseline characteristics and treatment in patients with disease control under chemotherapy with or without add-on nivolumab**

| **Characteristics** | | **Group B1** | **Group A** |  |
| --- | --- | --- | --- | --- |
| N | | 43 | 301 | P |
| Age (y/o) | median  range | 65  37-78 | 65  28-87 | 0.717 |
| Sex | male  female | 30  13 | 181  120 | 0.225 |
| Stage at diagnosis | I  II  III  IV | 1  5  8  29 | 15  53  90  143 | 0.109 |
| ECOG PS^§^ | 0-1  ≥2 | 38  5 | 247  54 | 0.304 |
| Primary site in pancreas | head  body  tail | 26  6  11 | 160  104  37 | 0.006 |
| Locoregional tumor^§^ | Yes  No | 36  7 | 274  27 | 0.133 |
| Metastatic tumor^§^ | Yes  No | 33  10 | 213  88 | 0.416 |
| Metastatic organ^§^ | Liver  Peritoneum  Lung | 21  13  7 | 110  118  66 | 0.120  0.257  0.397 |
| Curative surgery^§^ | Yes  No | 7  36 | 103  198 | 0.018 |
| Prior RT^§^ | Yes  No | 3  40 | 12  289 | 0.369 |
| **Treatment regimens and responses** | | | | |
| Regimens with disease control | First line  Second line  Third line  Later line | 28/43  29/43  28/36  13/24 | 234/301  121/235  50/149  33/87 | 0.069  0.054  <0.001  0.153 |
| First-line regimen^¶^ | Monotherapy  Doublet  Triplet  Quadruplet | 4  22  17  0 | 60  155  85  1 | 0.256 |
| Second-line regimen^¶^ | Monotherapy  Doublet  Triplet  Quadruplet | 0  20  21  2 | 39  130  65  1 | <0.001 |

^§^Before nivolumab treatment (group B1) and before the first-line chemotherapy for disease control (group A).

^¶^Considering only patients who received chemotherapy with/without targeted therapy.

ECOG PS, Eastern Cooperative Oncology Group performance status; F, 5-FU/5-FU analog; Gem, gemcitabine; Iri, (liposomal) irinotecan; NA, not analyzed; Pac, (nab)-paclitaxel; Pt, platinum (oxaliplatin or cisplatin)
