# Supplementary material for: Adding-on nivolumab to chemotherapy-stabilized patients is associated with improved survival in advanced pancreatic ductal adenocarcinoma
Source: Cancer Immunol Immunother. 2024 Sep 9;73(11):227. doi: 10.1007/s00262-024-03821-3 (PMC11383886; doi:10.1007/s00262-024-03821-3)
Supplement: Supplementary file 7 — Supplementary file7 (DOCX 20 KB) [file 262_2024_3821_MOESM7_ESM.docx]

**Supplementary Table 3 Regimens used in group A**

|  | **First line** | | **Second line** | |
| --- | --- | --- | --- | --- |
| Regimen^†^ | Drug | N | Drug | N |
| Monotherapy | S ± L  G  C | 43  16  1 | S  G  C  Cap  F/L | 19  11  6  2  1 |
| Doublet | G/F/L  G/Cap  G/S ± L  G/C  G/O  G/N | 2  1  73  5  3  72 | G/S  G/C  G/O  G/N  Nal/F ± L  Nal/S  I/F/L  I/S  O/S  O/F/L  C/F/L  S/Cy  P/F/L  P/S  P/N | 25  5  5  28  34  2  7  4  5  3  1  7  2  1  1 |
| Triplet | G/O/S ± L  G/O/F/L  G/C/S/L  G/N/S ± L  G/N/Bev  G/N/SGT53  G/N/PEGPH20  (m)FFX  N/O/S/L  N/F/L/olaparib | 39  6  2  2  1  4  2  24  3  1 | G/O/S ± L  G/O/F/L  G/O/Cap  G/C/S/L  G/N/S ± L  G/N/Cy  G/S/Cy  G/O/Bev  (m)FFX  N/O/F/L  Nal/O/F/L  Nal/F/L/trametinib  I/C/F/L | 24  4  1  5  11  4  7  1  3  1  2  1  1 |
| Quadruplet | G/F/L/P/Bev | 1 | G/O/S/L/Cy | 1 |

†Leucovorin (L) itself is not regarded as an anticancer drug.

Bev, bevacizumab; Cap, capecitabine; C, cisplatin; Cy, cyclophosphamide; F, 5-FU; G, gemcitabine; I, irinotecan; L, leucovorin; (m)FFX, (modified) FOLFIRINOX; N, nab-paclitaxel; O, oxaliplatin; P, paclitaxel; S, S-1
