# Supplementary material for: Adding-on nivolumab to chemotherapy-stabilized patients is associated with improved survival in advanced pancreatic ductal adenocarcinoma
Source: Cancer Immunol Immunother. 2024 Sep 9;73(11):227. doi: 10.1007/s00262-024-03821-3 (PMC11383886; doi:10.1007/s00262-024-03821-3)
Supplement: Supplementary file 8 — Supplementary file8 (DOCX 30 KB) [file 262_2024_3821_MOESM8_ESM.docx]

**Supplementary Table 4 Baseline characteristics after propensity score matching**

| **Matching group** | | **First line** | | | **Second line** | | | **Subsequent line** | | |
| --- | --- | --- | --- | --- | --- | --- | --- | --- | --- | --- |
| **Group** | | **B1** | **A** | **P1** | **B1** | **A** | **P2** | **B1** | **A** | **P3** |
| N (matched) | | 19 | 19 |  | 8 | 8 |  | 13 | 13 |  |
| **Characteristics** | |  |  |  |  |  |  |  |  |  |
| Age (y/o)^∏^ | median  range | 64  49-78 | 58  46-79 | 0.854 | 67  47-74 | 68  50-78 | 0.603 | 65  37-76 | 67  43-71 | 0.474 |
| Sex | male  female | 10  9 | 6  13 | 0.189 | 5  3 | 5  3 | 1.000 | 12  1 | 4  9 | 0.004 |
| Stage at initial diagnosis | I  II  III  IV | 0  1  5  13 | 0  1  5  13 | 1.000 | 0  1  2  5 | 0  1  2  5 | 1.000 | 1  2  1  9 | 1  1  5  6 | 0.308 |
| Primary site in pancreas | head  body  tail | 10  4  5 | 11  2  6 | 0.669 | 4  1  3 | 5  2  1 | 0.486 | 10  1  2 | 6  6  1 | 0.086 |
| ECOG PS^‡^ | 0-1  ≥2 | 18  1 | 18  1 | 1.000 | 6  2 | 6  2 | 1.000 | 12  1 | 11  2 | 1.000 |
| Locoregional disease^‡^ | Yes  No | 16  3 | 16  3 | 1.000 | 7  1 | 7  1 | 1.000 | 12  1 | 12  1 | 1.000 |
| Metastasis^‡^ | Yes  No | 13  6 | 14  5 | 0.721 | 6  2 | 6  2 | 1.000 | 12  1 | 12  1 | 1.000 |
| Metastatic organ^‡^ | Liver  Peritoneum  Lung | 10  6  4 | 8  8  4 | 0.516  0.501  1.000 | 3  2  1 | 2  1  2 | 1.000  1.000  1.000 | 8  3  2 | 5  5  4 | 0.239  0.673  0.645 |
| Curative surgery^‡^ | Yes  No | 2  17 | 3  16 | 1.000 | 1  7 | 1  7 | 1.000 | 3  10 | 5  8 | 0.673 |
| Radiotherapy to primary site^‡^ | Yes  No | 1  18 | 2  17 | 1.000 | 1  7 | 1  7 | 1.000 | 1  12 | 0  13 | 1.000 |
| Prior used chemotherapy agents^‡^ | Gem  F  Pt  Pac  Iri | 1  2  0  0  0 | 1  3  0  0  0 | 1.000  1.000  NA  NA  NA | 6  6  4  2  2 | 7  5  3  3  1 | 1.000  1.000  1.000  1.000  1.000 | 13  12  8  7  5 | 13  12  9  7  3 | NA  1.000  1.000  1.000  0.673 |
| First line regimen^†^ | Mono  Doublet  Triplet | 1  9  9 | 3  11  5 | 0.310 | 1  4  3 | 0  6  2 | 0.449 | 2  8  3 | 2  8  3 | 1.000 |
| Second line regimen^†^ | Mono  Doublet  Triplet  Quadruplet | 0  11  8  0 | 3  7  3  0 | 0.074 | 0  5  3  0 | 0  3  5  0 | 0.619 | 3  8  2  0 | 3  9  1  0 | 0.822 |
| First-line Gem-based therapy | Yes  No | 17  2 | 15  4 | 0.660 | 5  3 | 7  1 | 0.569 | 12  1 | 11  2 | 1.000 |
| Failure of first line therapy | Imaging  Clinical  Death  AE | 11  1  0  0 | 11  4  0  2 | 0.220 | NA | NA | NA | NA | NA | NA |
| Failure of second line therapy | Imaging  Clinical  Death  AE | NA | NA | NA | 5  0  1  0 | 3  1  0  2 | 0.212 | NA | NA | NA |
| Failure of later line therapy | Imaging  Clinical  Death  AE | NA | NA | NA | NA | NA | NA | 10  0  1  0 | 5  4  0  1 | 0.054 |

^∏^t test

^‡^Status before respective line of matching

^†^Only considering chemotherapy and targeted agents

AE, adverse event; ECOG PS, Eastern Cooperative Oncology Group performance status; F, 5-FU/5-FU analog; Gem, gemcitabine; Iri, (liposomal) irinotecan; NA, not analyzed; Pac, (nab)-paclitaxel; Pt, platinum (oxaliplatin or cisplatin)
