# Supplementary material for: Adding-on nivolumab to chemotherapy-stabilized patients is associated with improved survival in advanced pancreatic ductal adenocarcinoma
Source: Cancer Immunol Immunother. 2024 Sep 9;73(11):227. doi: 10.1007/s00262-024-03821-3 (PMC11383886; doi:10.1007/s00262-024-03821-3)
Supplement: Supplementary file 9 — Supplementary file9 (DOCX 28 KB) [file 262_2024_3821_MOESM9_ESM.docx]

**Supplementary Table 5 Baseline characteristics after propensity score matching in patients without CIK cell therapy**

| **Matching group** | | **First line** | | | **Second line** | | | **Subsequent line** | | |
| --- | --- | --- | --- | --- | --- | --- | --- | --- | --- | --- |
| **Group** | | **B1** | **A** | **P1** | **B1** | **A** | **P2** | **B1** | **A** | **P3** |
| N (matched) | | 10 | 10 |  | 6 | 6 |  | 7 | 7 |  |
| **Characteristics** | |  |  |  |  |  |  |  |  |  |
| Age (y/o)^∏^ | median  range | 65  50-78 | 63  47-73 | 0.408 | 67  55-73 | 67  66-71 | 0.477 | 66  37-73 | 59  43-69 | 0.644 |
| Sex | male  female | 3  7 | 1  9 | 0.582 | 4  2 | 5  1 | 1.000 | 7  0 | 6  1 | 1.000 |
| Stage at initial diagnosis | I  II  III  IV | 0  0  3  7 | 0  0  3  7 | 1.000 | 0  1  1  4 | 0  0  1  5 | 0.574 | 1  1  0  5 | 1  0  2  4 | 0.375 |
| Primary site in pancreas | head  body  tail | 7  1  2 | 8  1  1 | 0.819 | 3  1  2 | 4  1  1 | 0.788 | 5  1  1 | 2  2  3 | 0.270 |
| ECOG PS^‡^ | 0-1  ≥2 | 9  1 | 9  1 | 1.000 | 5  1 | 5  1 | 1.000 | 6  1 | 5  2 | 1.000 |
| Locoregional disease^‡^ | Yes  No | 10  0 | 10  0 | 1.000 | 5  1 | 6  0 | 1.000 | 6  1 | 7  0 | 1.000 |
| Metastasis^‡^ | Yes  No | 7  3 | 7  3 | 1.000 | 5  1 | 5  1 | 1.000 | 7  0 | 7  0 | 1.000 |
| Metastatic organ^‡^ | Liver  Peritoneum  Lung | 5  4  4 | 4  5  3 | 1.000  1.000  1.000 | 2  2  1 | 2  2  1 | 1.000  1.000  1.000 | 3  3  2 | 5  4  1 | 0.592  1.000  1.000 |
| Curative surgery^‡^ | Yes  No | 0  10 | 0  10 | 1.000 | 1  5 | 0  6 | 1.000 | 2  5 | 3  4 | 1.000 |
| Radiotherapy to primary site^‡^ | Yes  No | 0  10 | 0  10 | 1.000 | 1  5 | 1  5 | 1.000 | 0  7 | 1  6 | 1.000 |
| Prior used chemotherapy agents^‡^ | Gem  F  Pt  Pac  Iri | 0  0  0  0  0 | 0  0  0  0  0 | 1.000  1.000  1.000  1.000  1.000 | 5  4  3  2  1 | 3  5  0  1  0 | 0.545  1.000  0.182  1.000  1.000 | 7  7  5  2  1 | 7  7  4  1  2 | 1.000  1.000  1.000  1.000  1.000 |
| First line regimen^†^ | Mono  Doublet  Triplet | 0  6  4 | 0  5  5 | 1.000 | 1  3  2 | 3  3  0 | 0.223 | 1  4  2 | 1  4  2 | 1.000 |
| Second line regimen^†^ | Mono  Doublet  Triplet  Quadruplet | 0  7  3  0 | 1  3  4  1 | 0.296 | 0  4  2  0 | 0  4  2  0 | 1.000 | 0  1  5  1 | 2  2  3  0 | 0.280 |

^∏^t test

^‡^Status before respective line of matching

^†^Only considering chemotherapy and targeted agents

AE, adverse event; CIK, cytokine-induced killer; ECOG PS, Eastern Cooperative Oncology Group performance status; F, 5-FU/5-FU analog; Gem, gemcitabine; Iri, (liposomal) irinotecan; NA, not analyzed; Pac, (nab)-paclitaxel; Pt, platinum (oxaliplatin or cisplatin)
