# Supplementary material for: Adding-on nivolumab to chemotherapy-stabilized patients is associated with improved survival in advanced pancreatic ductal adenocarcinoma
Source: Cancer Immunol Immunother. 2024 Sep 9;73(11):227. doi: 10.1007/s00262-024-03821-3 (PMC11383886; doi:10.1007/s00262-024-03821-3)
Supplement: Supplementary file 10 — Supplementary file10 (DOCX 18 KB) [file 262_2024_3821_MOESM10_ESM.docx]

**Supplementary Table 6 Survival of propensity score-matched patients without CIK cell therapy**

|  | **Group B1** | **Group A** | **P** |
| --- | --- | --- | --- |
| First-line matching (n = 20) | | | |
| Median OS (month)  (95% CI) | 20.9  (NA) | 13.3  (0–27.4) | 0.284 |
| Median TTF (month)  (95% CI) | 12.1  (10.5–13.8) | 5.4  (3.4–7.5) | 0.101 |
| Second-line matching (n = 12) | | | |
| Median OS (month)  (95% CI) | 12.3  (0–31.3) | 8.6  (3.6–13.6) | 0.101 |
| Median TTF (month)  (95% CI) | 11.4  (0–31.3) | 5.4  (3.4–7.5) | 0.096 |
| Subsequent-line (not matched; n = 14) | | | |
| Median OS (month)  (95% CI) | 12.2  (7.9–16.5) | 12.0  (0–26.8) | 0.577 |
| Median TTF (month)  (95% CI) | 7.4  (4.9–9.9) | 4.8  (3.4–6.1) | 0.581 |
| Overall (n = 46) | | | |
| Median OS (month)  (95% CI) | 17.0  (10.1–23.9) | 12.0  (9.8–14.2) | 0.077 |
| Median TTF (month)  (95% CI) | 11.2  (7.2–15.2) | 5.4  (4.2–6.7) | 0.007 |

CI, confidence interval; CIK, cytokine-induced killer; OS, overall survival; TTF, time to treatment failure
