# Supplementary material for: Adding-on nivolumab to chemotherapy-stabilized patients is associated with improved survival in advanced pancreatic ductal adenocarcinoma
Source: Cancer Immunol Immunother. 2024 Sep 9;73(11):227. doi: 10.1007/s00262-024-03821-3 (PMC11383886; doi:10.1007/s00262-024-03821-3)
Supplement: Supplementary file 11 — Supplementary file11 (DOCX 18 KB) [file 262_2024_3821_MOESM11_ESM.docx]

**Supplementary Table 7 Common and DDR-associated mutations in nivolumab-treated patients**

| **Gene** | ***KRAS*** | ***TP53*** | ***CDKN2A*** | ***SMAD4*** | ***ATM*** | ***POLE*** |
| --- | --- | --- | --- | --- | --- | --- |
| N of tested | 42 | 39 | 39 | 39 | 39 | 39 |
| N of alterations | 36 (86%) | 23 (59%) | 10 (26%) | 6 (15%) | 4 (10%) | 2 (5%) |
| Alterations | G12D: 13  G12V: 12  G12R: 4  G12L: 1  G12H: 1  G13D: 1  Q61R: 2  Q61H: 1  Q61L: 1 | R110*: 1  Y126H: 1  P128fs: 1  C135Y: 1  Q144*: 1  G154_T155 del: 1  R156fs: 1  I162N: 1  R175H: 2  H179L: 1  H179Y: 1  R196*: 1  R213*: 1  R248Q: 1  R248W: 1  G266E: 1  R273C: 1  R273H: 1  V274L: 1  R280G: 1  S303fs: 1  R306*: 1 | Homozygous deletion: 7  R58*: 1  H83Y: 1  D84G: 1 | Homozygous deletion: 2  E31fs: 1  S242*: 1  A258fs: 1  Y301fs: 1 | C1899*: 1  I2629fs: 1  E2711*: 1  L3010_R3012delins*: 1 | R52fs: 1  R2127*: 1 |
| **Gene** | ***CHEK2*** | ***FANCC*** | ***MSH2*** | ***MSH6*** | ***ERCC1*** | ***BRCA2*** |
| N of tested | 39 | 39 | 39 | 39 | 39 | 39 |
| N of alterations | 1 (3%) | 1 (3%) | 1 (3%) | 1 (3%) | 1 (3%) | 1 (3%) |
| Alterations | H371Y: 1 | W113*: 1 | Homozygous deletion: 1 | Y1006*: 1 | Amplification: 1 | N588fs: 1 |

DDR, DNA damage response
