# Supplementary material for: Adding-on nivolumab to chemotherapy-stabilized patients is associated with improved survival in advanced pancreatic ductal adenocarcinoma
Source: Cancer Immunol Immunother. 2024 Sep 9;73(11):227. doi: 10.1007/s00262-024-03821-3 (PMC11383886; doi:10.1007/s00262-024-03821-3)
Supplement: Supplementary file 12 — Supplementary file12 (DOCX 19 KB) [file 262_2024_3821_MOESM12_ESM.docx]

**Supplementary Table 8 Patients with immune profiling and IHC analysis**

| **Case** | **Age** | **Sex** | **Stage** | **TTS**  **(month)** | **RT** | **OS_chemo_**  **(month)** | **Status** |
| --- | --- | --- | --- | --- | --- | --- | --- |
| Group B1 | | | | | | | |
| B1-1 | 66 | F | 3 | 7.3 | - | 21.2 | live |
| B1-2 | 52 | M | 4 | 7.0 | - | 20.0 | dead |
| B1-3 | 64 | F | 4 | 6.2 | - | 28.9 | live |
| Group A | | | | | | | |
| A-1 | 71 | F | 3 | 7.3 | - | 50.0 | live |
| A-2 | 54 | M | 3 | 5.1 | + | 47.6 | live |
| A-3 | 69 | F | 3 | 7.3 | - | 36.6 | live |
| A-4 | 63 | M | 3 | 6.0 | - | 39.0 | live |
| A-5 | 71 | M | 3 | 4.4 | - | 31.5 | dead |
| A-6 | 43 | F | 3 | 6.3 | - | 45.4 | live |
| A-7 | 71 | M | 3 | 4.8 | - | 27.0 | dead |
| A-8 | 55 | M | 3 | 5.8 | + | 9.8 | dead |
| A-9 | 78 | F | 3 | 2.9 | - | 21.0 | live |

TTS, time (first-line therapy) to surgery; RT, radiotherapy (before surgery); OS_chemo_, overall survival since the initiation of chemotherapy achieving disease control
